# Treatment of node-positive endometrial cancer with complete node dissection, chemotherapy and radiation therapy.

**DOI:** 10.1038/bjc.1997.313

**Published:** 1997

**Authors:** T. Onda, H. Yoshikawa, K. Mizutani, M. Mishima, H. Yokota, H. Nagano, Y. Ozaki, A. Murakami, K. Ueda, Y. Taketani

**Affiliations:** Department of Obstetrics and Gynecology, Faculty of Medicine, University of Tokyo, Bunkyo-ku, Japan.

## Abstract

We assessed the therapeutic significance of systematic aortic and pelvic lymphadenectomy followed by adjuvant therapy in node-positive endometrial carcinoma. Among 173 stage I-III patients, 30 (17%) had positive nodes: ten in the pelvic region alone (group P) and 20 in the aortic region alone or in both regions (group A). The adjuvant therapy was administered as follows: subjects in group P received 50 Gy pelvic radiation, including three post-surgical T3 (pT3) patients who received either one or three cycles of cisplatin-based chemotherapy before radiation. Subjects in group A were given three cycles of chemotherapy followed by 50 Gy pelvic and 50 Gy extended field periaortic radiation using a four-field or conformational technique. Five-year survival was 95% for 143 patients with negative nodes and 84% for 30 patients with positive nodes (100% for group P and 75% for group A). In group A, 5-year survival was 38% for eight patients with both pT3 and histology other than endometrioid type G1, and 91% for the remaining 12 patients. Either way, both group P and group A patients had a better prognosis than previously reported. In summary, aortic and pelvic lymphadenectomy and subsequent chemotherapy and radiation therapy based on node status seem to improve the survival of endometrial cancer patients with positive nodes.


British Journal of Cancer (1997) 75(12), 1836–1841
© 1997 Cancer Research Campaign

### Treatment of node-positive endometrial cancer with complete node dissection, chemotherapy and radiation therapy

**T Onda[1], H Yoshikawa[1], K Mizutani[2], M Mishima[1], H Yokota[1], H Nagano[2], Y Ozaki[2], A Murakami[2], K Ueda[2] and Y Taketani[1]**

[1]Department of Obstetrics and Gynecology, Faculty of Medicine, University of Tokyo, Tokyo, Japan; [2]Department of Obstetrics and Gynecology, Tokyo Metropolitan Komagome Hospital, Tokyo, Japan

**Summary** We assessed the therapeutic significance of systematic aortic and pelvic lymphadenectomy followed by adjuvant therapy in node-positive endometrial carcinoma. Among 173 stage I–III patients, 30 (17%) had positive nodes: ten in the pelvic region alone (group P) and 20 in the aortic region alone or in both regions (group A). The adjuvant therapy was administered as follows: subjects in group P received 50 Gy pelvic radiation, including three post-surgical T3 (pT3) patients who received either one or three cycles of cisplatin-based chemotherapy before radiation. Subjects in group A were given three cycles of chemotherapy followed by 50 Gy pelvic and 50 Gy extended field periaortic radiation using a four-field or conformational technique. Five-year survival was 95% for 143 patients with negative nodes and 84% for 30 patients with positive nodes (100% for group P and 75% for group A). In group A, 5-year survival was 38% for eight patients with both pT3 and histology other than endometrioid type G1, and 91% for the remaining 12 patients. Either way, both group P and group A patients had a better prognosis than previously reported. In summary, aortic and pelvic lymphadenectomy and subsequent chemotherapy and radiation therapy based on node status seem to improve the survival of endometrial cancer patients with positive nodes.

**Keywords:** endometrial cancer; lymph node metastasis; aortic and pelvic lymphadenectomy; adjuvant therapy

Based on the FIGO (the International Federation of Gynecology and Obstetrics) staging system proposed in 1988, patients with positive aortic or pelvic nodes are classified as having stage IIIC disease (Creasman, 1990), which is associated with the poorest prognosis among patients without distant metastases. The survival of stage IIIC patients ranges from 0% to 56% throughout the literature (Potish et al, 1985; Larson et al, 1987; Greven et al, 1993).

Various procedures have been used to assess aortic and pelvic nodes in endometrial cancer, i.e. biopsies from enlarged nodes only, selective nodal sampling from multiple sites, pelvic lymphadenectomy and aortic and pelvic lymphadenectomy. It is obvious that aortic and pelvic lymphadenectomy is most accurate of these methods. However, aortic and pelvic lymphadenectomy is not regarded as the standard surgical procedure for endometrial cancer, even among patients at high risk for lymph node metastases, because the therapeutic significance of the procedure has not yet been sufficiently demonstrated (Calais et al, 1990; Belinson et al, 1992; Kim et al, 1993; Faught et al, 1994; Chuang et al, 1995; Kilgore et al, 1995).

Feuer et al (1987) and Rose et al (1992) reported that post-surgical radiotherapy was effective for microscopic node metastases of endometrial cancer, whereas it had no favourable effect on macroscopic node metastases. On the other hand, the response rate of chemotherapy for endometrial cancer has been reported to be 30–57% (Seski et al, 1982; Hancock et al, 1986; Green et al, 1990), which is still lower than that for ovarian cancer. Hence, the rational treatment of endometrial cancer with positive lymph nodes is not clarified.

In an attempt to improve treatment results of endometrial cancer patients with positive lymph nodes, we examined the therapeutic value of systematic aortic and pelvic lymphadenectomy followed by radiation therapy or chemotherapy plus radiation therapy, depending on node status. We also investigated the incidence and distribution of node metastases in relation to various prognostic variables of endometrial cancer.

*Received 22 October 1996*
*Revised 24 December 1996*
*Accepted 18 January 1997*

*Correspondence to:* T Onda, Department of Obstetrics and Gynecology, Faculty of Medicine, University of Tokyo, Hongo 7-3-1, Bunkyo-ku, Tokyo 113, Japan

### PATIENTS AND METHODS

### Patient selection

From July 1986 to March 1996, we performed systematic aortic and pelvic lymphadenectomy in addition to hysterectomy and bilateral salpingo-oophorectomy in 173 stage I–III endometrial cancer patients treated at the Department of Obstetrics and Gynecology, University of Tokyo Hospital, or at the Department of Obstetrics and Gynecology, the Tokyo Metropolitan Komagome Hospital. The mean age of the 173 patients was 55.8 years (range 30–77 years), with the median follow-up being 50 months (range 4–111 months), exclusive of death cases.

Histological type and grade, myometrial invasion and post-surgical T (pT) classification of the 173 patients are shown in Table 1. Definition of stage and TNM classification are as follows: stage I (pT1N0M0); tumour confined to the corpus (T1), no evidence of lymph node metastasis (N0), no evidence of distant metastasis

**Table 1** Histological type and grade, myometrial invasion and pT classification of the stage I–III patients who underwent aortic and pelvic lymphadenectomy

| Histological type and grade | No. of patients | pT classification | | |
|---|---|---|---|---|
| | | pT1 | pT2 | pT3 |
| Endometrioid | | | | |
| G1 | 97 | 63 | 15 | 19 |
| G2 | 55 | 33 | 6 | 16 |
| G3 | 10 | 6 | 1 | 3 |
| Adenosquamous | 9 | 3 | 4 | 2 |
| Clear cell | 1 | 0 | 0 | 1 |
| Squamous | 1 | 1 | 0 | 0 |
| Total | 173 | 106 | 26 | 41 |
| Myometrial invasion | | | | |
| < 1/3 | 93 | 66 | 11 | 16 |
| 1/3–2/3 | 35 | 21 | 7 | 7 |
| > 2/3 | 45 | 19 | 8 | 18 |
| Total | 173 | 106 | 26 | 41 |

(M0); stage II (pT2N0M0), tumour invades the cervix but does not extend beyond the uterus (T2); stage IIIA (pT3AN0M0), tumour invades serosa and/or adnexa (direct extension or metastasis) and/or cancer cells in ascites or peritoneal washings (T3A); stage IIIB (pT3BN0M0), vaginal involvement (direct extension or metastasis) (T3B); stage IIIC (pT1–3N1M0), evidence of lymph node metastasis (N1).

### Surgery

Operative procedure included hysterectomy, bilateral salpingo-oophorectomy and systematic aortic and pelvic lymphadenectomy.

Radical hysterectomy was performed on 66 patients with positive findings on presurgical endocervical curettage and total hysterectomy on the remainder, who were negative for endocervical curettage.

The procedure of systematic lymphadenectomy performed in this study represents complete dissection of lymph nodes from the femoral ring caudally up to the upper margin of the renal vessels. To achieve this, we mobilized the ascending colon, the descending colon and the duodenum, and displaced them to the right, to the left and upwards respectively, so that para-aortic retroperitoneal space was widely developed up to the renal vessels. All lymphatic tissues surrounding the retroperitoneal vessels were completely removed. The average number (range) of nodes removed was 66.7 (37–96): 28.7 for the aortic nodes (10–49) and 37.9 for the pelvic nodes (23–57).

All 173 patients had no residual tumours after completion of surgery including the ablation of peritoneal implants.

### Chemotherapy

Post-surgically, the patients with either adnexal/peritoneal involvement or aortic node metastases were treated with three cycles of chemotherapy (CAP regimen) at 3 weeks' interval followed by radiation therapy. The patients who did not meet the above criteria and had positive peritoneal cytology were treated with one cycle of CAP. The CAP regimen consisted of cyclophosphamide 600 mg $m^{-2}$, doxorubicin 40 mg $m^{-2}$ and cisplatin 75 mg $m^{-2}$. Each anti-cancer drug was administered intravenously on the same day.

**Table 2** Incidence of lymph node metastases in relation to various prognostic factors

| | No. of patients | Lymph node metastases | | |
|---|---|---|---|---|
| | | Pelvic (%) | Aortic (%) | Total (%) |
| pT classification | | | | |
| pT1 | 106 | 10 (9%) | 4 (4%) | 10 (9%) |
| pT2 | 26 | 4 (15%) | 4 (15%) | 5 (19%) |
| pT3[a] | 41 | 14 (34%) | 12 (29%) | 15 (37%) |
| Histological type and grade | | | | |
| Endometrioid | | | | |
| G1 | 97 | 8 (8%) | 5 (5%) | 9 (9%) |
| G2 | 55 | 15 (27%) | 12 (22%) | 16 (29%) |
| G3 | 10 | 1 (10%) | 0 (0%) | 1 (10%) |
| Adenosquamous | 9 | 3 (33%) | 2 (22%) | 3 (33%) |
| Clear cell | 1 | 1 (100%) | 1 (100%) | 1 (100%) |
| Squamous | 1 | 0 (0%) | 0 (0%) | 0 (0%) |
| Myometrial invasion | | | | |
| < 1/3 | 93 | 8 (9%) | 4 (4%) | 8 (9%) |
| 1/3–2/3 | 35 | 6 (17%) | 4 (11%) | 6 (17%) |
| > 2/3 | 45 | 14 (31%) | 12 (27%) | 16 (36%) |
| Overall | 173 | 28 (16%) | 20 (12%) | 30 (17%) |

[a]All 41 patients were categorized as pT3A.

### Radiation therapy

Whole-pelvis irradiation was indicated when at least one of the following factors existed: positive pelvic lymph nodes, deep myometrial invasion (more than two-thirds invasion in endometrioid G1, more than one-third in other types or grades), pelvic peritoneal metastases and adnexal metastases. The periaortic irradiation was administered to aortic node-positive patients.

Both pelvic and periaortic radiation treatments consisted of 50 Gy administered in 2-Gy fractions, five times per week for 5 weeks. The periaortic irradiation was administered with 10 MeV X-rays employing a conformational technique using computer planning or with a four-field [anterior–posterior (7 cm wide) and lateral (6 cm wide) fields] technique (Corn et al, 1992) from the upper margin of the fifth lumbar vertebral body to that of the 11th thoracic vertebral body. Both methods were planned to deliver 50-Gy dosages to aortic node region and 25 Gy or less in large part of peritoneal cavity and the bone marrow of lumbar and thoracic vertebral bodies. Radiotherapy was initiated 3 weeks after chemotherapy.

### Statistical methods

Survival curves were determined by the Kaplan–Meier product limit method (Kaplan et al, 1958). Analysis of the differences between survival curves was performed using the log-rank test. A multifactorial approach (Cox proportional-hazards regression analysis) was performed in analysing the prognostic factors using a JMP program.

### RESULTS

### Incidence and distribution of lymph node metastases

The overall incidence of retroperitoneal lymph node metastases assessed by systematic aortic and pelvic lymphadenectomy was 17% (30/173) in stage I–III endometrial cancer. The incidences of aortic and pelvic lymph node metastases were 12% (20/173) and

© Cancer Research Campaign 1997

16% (28/173) respectively (Table 2). More precisely, two patients had lymph node metastases in the aortic region alone, ten in the pelvic region alone and 18 in both regions.

We examined the incidences of aortic and pelvic node metastases in relation to pT classification, histological type and grade and depth of myometrial invasion (Table 2). The incidences of positive lymph nodes were 9% in pT1, 19% in pT2 and 37% in pT3. The incidences of positive aortic and pelvic nodes were, respectively, 4% and 9% in pT1, 15% and 15% in pT2 and 29% and 34% in pT3. As for histological type, the incidences of node metastases were 16% (26/162) in endometrioid adenocarcinoma, 33% (3/9) in adenosquamous carcinoma, 100% (1/1) in clear cell adenocarcinoma and 0% (0/1) in squamous cell carcinoma. In relation to grade of endometrioid adenocarcinoma, the incidences of node metastases were 9% (9/97) in G1, 29% (16/55) in G2 and 10% (1/10) in G3 tumours. The incidence of node metastases increased with depth of maximal myometrial invasion such that node metastases were found in 9% (8/93) of patients with the cancer limited to the endometrium or superficial myometrial invasion (inner one-third), 17% (6/35) of patients with intermediate

**Figure 1** Survival of patients with stage I/II, IIIA and IIIC endometrial cancer who underwent systematic lymphadenectomy

**Figure 2** Survival of stage IIIC endometrial cancer patients according to localization of the node metastases

© Cancer Research Campaign 1997

**Figure 3** Survival of aortic node-positive endometrial cancer patients with or without risk factors of both histology other than endometrioid G1 and pT3

myometrial invasion (middle one-third) and 36% (16/45) of patients with deep myometrial invasion (outer one-third).

### Adjuvant therapy of the node-positive patients

All 30 endometrial cancer patients with lymph node metastases were treated according to the treatment programme as described in 'Patients and methods'. As a result, 20 patients with aortic node metastases (group A) had both whole pelvis irradiation and periaortic irradiation after three cycles of CAP, because pelvic irradiation was administered in 18 patients for concomitant pelvic node metastases and in two patients for deep myometrial invasion.

Of the remaining ten patients with pelvic node metastases alone (group P), one patient had one cycle of CAP for positive peritoneal cytology and two had three cycles of CAP for adnexal metastases followed by whole-pelvis irradiation.

Looking at the accomplishment of the treatment, no patients required major modification of treatment modality because of acute complications such as myelosuppression and enteritis. One patient developed partial small bowel obstruction that required the surgery of affected bowel and reanastomosis 36 months after pelvic and periaortic irradiation.

### Survival

Five-year survival was 93% for all the 173 stage I–III patients. Figure 1 shows the survival data in relation to FIGO stage. Five-year survival of the patients with stage I/II, IIIA, IIIC was 95%, 96% and 84% respectively.

Survival of the patients with lymph node metastases (the stage IIIC patients) was analysed according to localization of the node metastases (Figure 2). Five-year survival was 75% for the patients with aortic node metastases irrespective of pelvic node metastases (group A) and 100% for those with pelvic node metastases alone (group P).

We further analysed correlation between the survival of group A patients and various prognostic factors such as pT classification, histology and grade and depth of myometrial invasion. Five-year survival was 88% for patients with pT1/2 disease and 62% for those with pT3 disease ($P$ = 0.30, not significant), 100% for the patients with endometrioid G1 cancer and 68% for those with histology other than endometrioid G1 ($P$ = 0.26, not significant), 75% for the patients with superficial myometrial invasion (less than one-third) and 75% for those with moderate to deep myometrial invasion (beyond one-third) ($P$ = 0.76, not significant). Multifactorial analysis revealed no significant correlation between survival in group A and each prognostic factor.

These results suggested that histology other than endometrioid G1 and pT3 seemed to be associated with poor prognosis in group A, although the differences were not statistically significant. Subsequently, we compared the survival curve for patients with these two prognostic factors (histology other than endometrioid G1 and pT3) with that for patients with one or none of these factors (Figure 3). Five-year survival among patients with both prognostic factors was significantly poorer than for those with one or no prognostic factor (38% vs 91%, $P < 0.05$).

### DISCUSSION

In this study, we analysed the prevalence and distribution of metastatic aortic and pelvic lymph nodes and the survival of endometrial cancer patients who underwent systematic aortic and pelvic lymphadenectomy followed by irradiation or chemotherapy plus irradiation, depending on node status.

The incidences of aortic and pelvic node metastases in stage I–III endometrial cancer were 12% (20/173) and 16% (28/173) respectively. Creasman et al (1987) (Gynecologic Oncology Group) reported the incidences of aortic and pelvic node metastases were 6% and 9% respectively. The incidences in the present study were higher than those in previous reports (aortic nodes,

© Cancer Research Campaign 1997

1–9%; pelvic nodes, 9–15%) (Burrell et al, 1982; Boronow et al, 1984; Ayhan et al, 1989). The reason for this may lie in differences in the subjects examined, such that previous studies dealt with patients with clinical (preoperative) stage I disease, whereas our study included patients with stage I–III cancer. In addition, we conducted lymph node dissection thoroughly, as reflected by an average number of removed nodes of 66.7. This may, in part, explain the high positive rates in our study.

Concerning the distribution of lymph node metastases, 93% (28/30) of patients with node metastases had pelvic node metastases and 64% (18/28) of patients with pelvic node metastases had concomitant aortic node metastases. The high incidence of pelvic node metastases accompanied by aortic node metastases is consistent with previous reports (Boronow et al, 1984). In contrast, 33% (10/30) of patients with node metastases had pelvic node metastases alone and 7% (2/30) had aortic node metastases alone. Creasman et al (1987) (GOG) also reported a higher incidence of isolated pelvic node metastases (51% [36/70]) than of isolated aortic node metastases (17% [12/70]) in clinical stage I endometrial cancer patients. These data suggest that in endometrial cancer direct lymphatic spread to pelvic nodes occurs more frequently than spread to the aortic nodes.

We correlated lymph node metastases with other prognostic factors such as pT, histological type and grade, and myometrial invasion. The incidence of lymph node metastases increased with advancement of pT and depth of myometrial invasion. The incidence of lymph node metastases was lower in endometrioid G1 than in endometrioid G2/G3 or other histological types. These findings are consistent with previous reports on clinical stage I/II endometrial cancer (Burrell et al, 1982; Boronow et al, 1984; Creasman et al, 1987; Ayhan et al, 1989; Calais et al, 1990; Morrow et al, 1991).

pT3A is defined as the presence of adnexal metastases, pelvic peritoneal implants and positive peritoneal cytology. Thus far, these clinical manifestations that are characteristic of pT3A are thought to be poor prognostic factors (Creasman et al, 1981; Morrow et al, 1991; Greven et al, 1993; Kadar et al, 1994). The present study demonstrated that 37% of pT3A patients had lymph node metastases. Notably, when focusing on stage IIIA patients in the pT3A group, we found a higher 5-year survival rate among the patients with stage IIIA disease (96%) than among those with stage I/II disease (95%). The good prognosis of the stage IIIA (pT3AN0) patients in our study can be explained as follows. Firstly, lymph node-positive patients (pT3AN1) could be completely excluded by systematic lymphadenectomy. Secondly, post-surgical chemotherapy with or without radiation therapy performed in this study may in part account for the favourable result. At any rate, this result is taken to imply that the setting associated with pT3A does not necessarily represent an unfavourable outcome.

Survival of patients with stage IIIC disease (84%) is much better than that reported previously (Potish et al, 1985; Larson et al, 1987; Greven et al, 1993). We analysed the survival of the patients with stage IIIC disease in relation to localization of node metastases and found that positive aortic nodes were associated with poorer prognosis than were positive pelvic nodes (75% vs. 100%). Although metastasis to pelvic nodes has been reported to be as poor a prognostic factor as metastasis to aortic nodes (41–67% 5-year survival) (Potish et al, 1985; Calais et al, 1990), the present study suggests that involvement of pelvic lymph nodes alone does not necessarily carry a poor prognosis. Kadar et al (1994) reported that the 5-year survival was 82% for patients with pelvic node metastases alone as assessed by pelvic and aortic lymphadenectomy below the inferior mesenteric artery. In the present study, over 60% of patients with positive pelvic nodes had concomitant aortic node metastases, which could be an explanation for the notion that metastasis to pelvic nodes is a poor prognostic factor. The good prognosis of the patients with pelvic node metastases alone (group P) may be attributed not only to our treatment programme, but also to complete exclusion of the presence of aortic node metastases by aortic lymphadenectomy.

In the present study, 5-year survival of patients with aortic node metastases (group A) (75%) was much better than previously reported (0–60%) (Komaki et al, 1983; Potish et al, 1985; Blythe et al, 1986; Feuer et al, 1987; Larson et al, 1987; Corn et al, 1992; Rose et al, 1992; Hicks et al, 1993; Kadar et al, 1994). In particular, aortic node-positive patients with pT1/2 or endometrioid G1 cancer had a good prognosis (91% 5-year survival). Corn et al (1992) reported that significantly fewer recurrences occurred among aortic node-positive patients who underwent nodal debulking followed by extended field irradiation and thus suggested that new systemic treatments are needed to resolve the problem of distant failure of these patients. Our data suggest that aortic and pelvic lymphadenectomy followed by adjuvant chemotherapy and radiation therapy may improve the survival of the endometrial cancer patients with positive aortic nodes.

As for the patients with aortic node metastases (group A), both histology other than endometrioid G1 and pT3 were shown to be associated with poorer prognosis (38% 5-year survival). Hence, aortic node metastasis still carries a poor prognosis, especially when other prognostic factors such as histology other than endometrioid G1 and pT3 are also present.

We conclude that aortic and pelvic lymphadenectomy followed by chemotherapy and radiotherapy based on histological findings of node status increases survival of endometrial cancer patients with positive nodes. However, further challenging treatment programmes may be necessary for aortic node-positive patients, especially for those with poor prognostic factors such as histology other than endometrioid G1 and pT3.

### REFERENCES

Ayhan A, Yarali H, Urman B, Günalp S, Yüce K, Ayhan A and Havlioglu S (1989) Lymph node metastasis in early endometrial cancer. *Aust NZ J Obstet Gynaecol* **29**: 332–335

Belinson JL, Lee KR, Badger GJ, Pretorius MD and Jarrell MA (1992) Clinical stage I adenocarcinoma of the endometrium: Analysis of recurrences and potential benefit of staging lymphadenectomy. *Gynecol Oncol* **44**: 17–23

Blythe JG, Hodel KA, Wahl TP, Baglan RJ, Lee FA and Zivnuska FR (1986) Para-aortic node biopsy in cervical and endometrial cancers: Does it affect survival? *Am J Obstet Gynecol* **155**: 306–314

Boronow RC, Morrow CP, Creasman WT, Disaia PJ, Silverberg SG, Miller A and Blessing JA (1984) Surgical staging in endometrial cancer: Clinical–pathological findings of a prospective study. *Obstet Gynecol* **63**: 825–832

Burrell MO, Franklin III EW and Powell JL (1982) Endometrial cancer: evaluation of spread and follow-up in one hundred eighty-nine patients with stage I or stage II disease. *Am J Obstet Gynecol* **144**: 181–185

Calais G, Descamps P, Vitu L, Body G, Lansac J, Bougnoux P and Floch OL (1990) Is lymphadenectomy useful in the treatment of endometrial carcinoma? *Gynecol Oncol* **38**: 71–75

Chuang L, Burke TW, Tornos C, Marino BD, Mitchell MF, Tortolero-Luna G, Levenback C, Morris M and Gershenson DM (1995) Staging laparotomy for endometrial carcinoma: Assessment of retroperitoneal lymph nodes. *Gynecol Oncol* **58**: 189–193

© Cancer Research Campaign 1997

Corn BW, Lanciano RM, Greven KM, Schultz DJ, Reisinger SA, Stafford PM and Hanks GE (1992) Endometrial cancer with para-aortic adenopathy: Patterns of failure and opportunities for cure. *Int J Radiation Oncology Biol Phys* **24**: 223–227

Creasman WT (1990) New gynecologic cancer staging. *Obstet Gynecol* **75**: 287–288

Creasman WT, Disaia PJ, Blessing J, Wilkinson JR RH, Johnston W and Weed Jr JC (1981) Prognostic significance of peritoneal cytology in patients with endometrial cancer and preliminary data concerning therapy with intraperitoneal radiopharmaceuticals. *Am J Obstet Gynecol* **141**: 921–929

Creasman WT, Morrow CP, Bundy BN, Homesley HD, Graham JE and Heller PB (1987) Surgical pathologic spread patterns of endometrial cancer. A Gynecologic Oncology Group Study. *Cancer* **60**: 2035–2041

Faught W, Krepart GV, Lotocki R and Heywood M (1994) Should selective paraaortic lymphadenectomy be part of surgical staging for endometrial cancer? *Gynecol Oncol* **55**: 51–55

Feuer GA and Calanog A (1987) Endometrial carcinoma: Treatment of positive paraaortic nodes. *Gynecol Oncol* **27**: 104–109

Green III JB, Green S, Albert DS, O'Toole R, Surwit EA and Noltimier JW (1990) Carboplatin therapy in advanced endometrial cancer. *Obstet Gynecol* **75**: 696–700

Greven KM, Lanciano RM, Corn B, Case D and Randall ME (1993) Pathologic stage III endometrial carcinoma. Prognostic factors and patterns of recurrence. *Cancer* **71**: 3697–3702

Hancock KC, Freedman RS, Edwards CL and Rutledge FN (1986) Use of cisplatin, doxorubicin, and cyclophosphamide to treat advanced and recurrent adenocarcinoma of the endometrium. *Cancer Treat Rep* **70**: 789–791

Hicks ML, Piver MS, Puretz JL, Hempling RE, Baker TR, McAuley M and Walsh DL (1993) Survival in patients with paraaortic lymph node metastases from endometrial adenocarcinoma clinically limited to the uterus. *Int J Radiation Oncology Biol Phys* **26**: 607–611

Kadar N, Homesley HD and Malfetano JH (1994) Prognostic factors in surgical stage III and IV carcinoma of the endometrium. *Obstet Gynecol* **84**: 983–986

Kaplan EL and Meier P (1958) Nonparametric estimation from incomplete observations. *J Am Stat Assoc* **53**: 457–481

Kilgore LC, Partridge EE, Alvarez RD, Austin JM, Shingleton HM, Noojin III F and Conner W (1995) Adenocarcinoma of the endometrium: Survival comparisons of patients with and without pelvic node sampling. *Gynecol Oncol* **56**: 29–33

Kim YB and Niloff JM (1993) Endometrial carcinoma: Analysis of recurrence in patients treated with a strategy minimising lymph node sampling and radiation therapy. *Obstet Gynecol* **82**: 175–180

Komaki R, Mattingly RF, Hoffman RG, Barber SW, Satre R and Greenberg M (1983) Irradiation of para-aortic lymph node metastases from carcinoma of the cervix or endometrium. *Radiology* **147**: 245–248

Larson DM, Copeland LJ, Gallager HS, Wharton JT, Gershenson DM, Edwards CL, Malone Jr JM and Rutledge FN (1987) Prognostic factors in stage II endometrial carcinoma. *Cancer* **60**: 1358–1361

Morrow CP, Bundy BN, Kurman RJ, Creasman WT, Heller P, Homesley HD and Graham JE (1991) Relationship between surgical-pathological risk factors and outcome in clinical stage I and II carcinoma of the endometrium: A Gynecologic Oncology Group Study. *Gynecol Oncol* **40**: 55–65

Potish RA, Twiggs LB, Adcock LL, Savage JE, Levitt SH and Prem KA (1985) Paraaortic lymph node radiotherapy in cancer of the uterine corpus. *Obstet Gynecol* **65**: 251–256

Rose PG, Cha SD, Tak WK, Fitzgerald T, Reale F and Hunter RE (1992) Radiation therapy for surgically proven para-aortic node metastasis in endometrial carcinoma. *Int J Rad Oncol Biol Phys* **24**: 229–233

Seski JC, Edwards CL, Herson J and Rutledge FN (1982) Cisplatin chemotherapy for disseminated endometrial cancer. *Obstet Gynecol* **59**: 225–228

© Cancer Research Campaign 1997


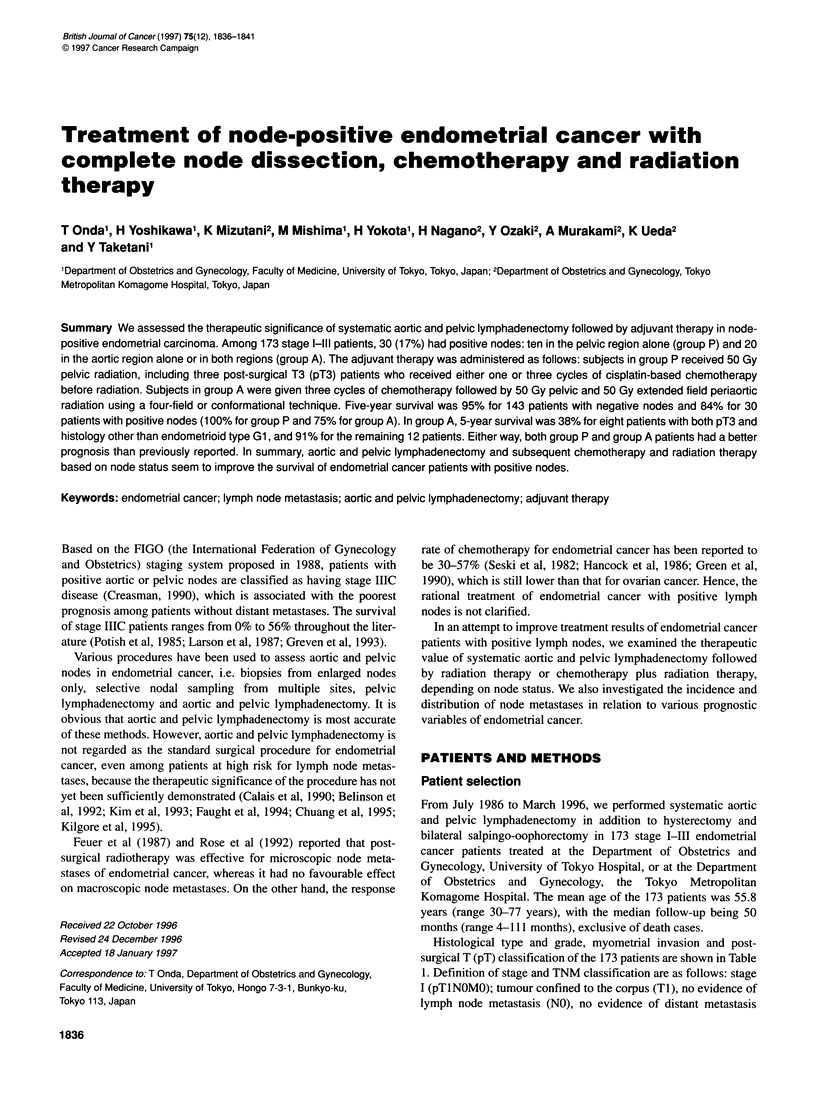

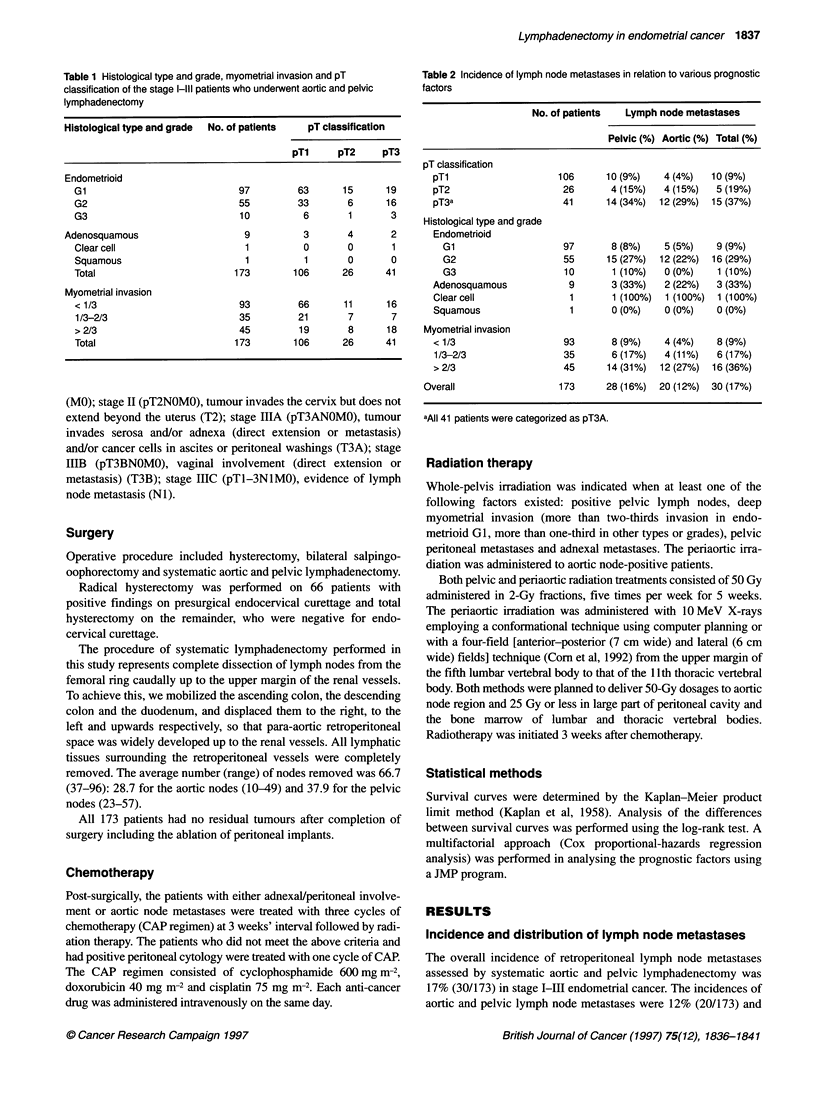

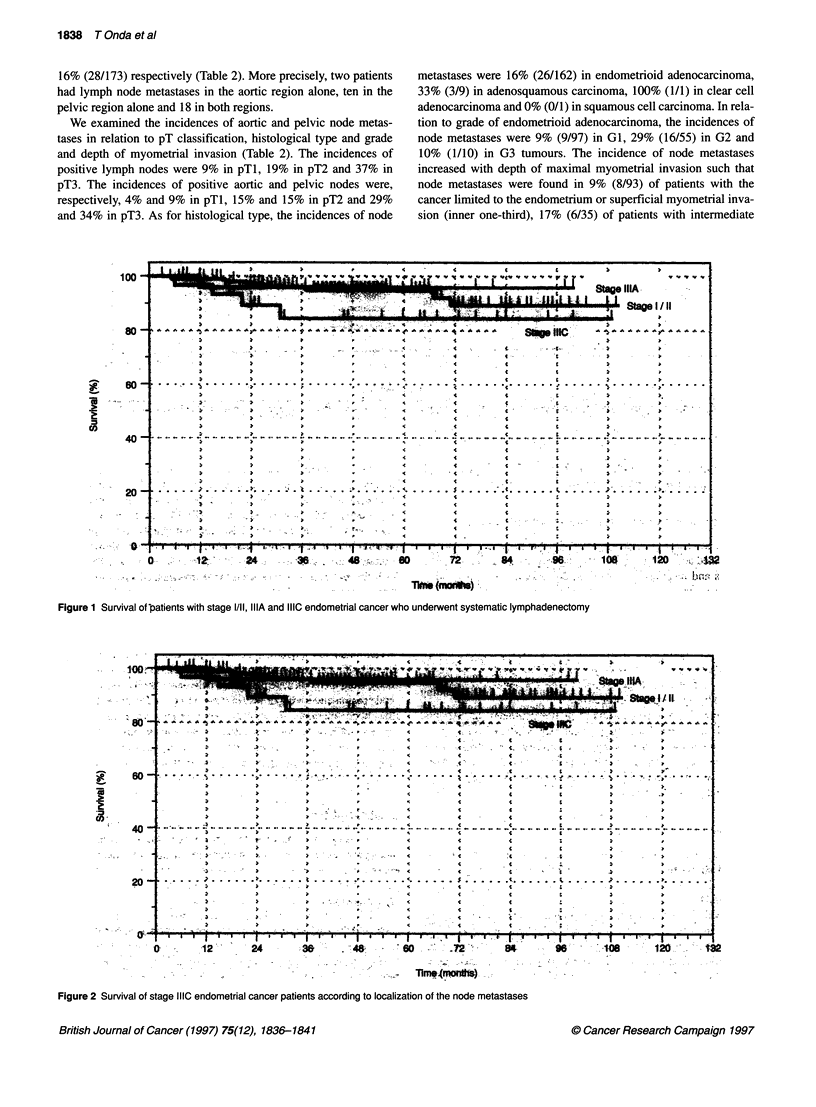

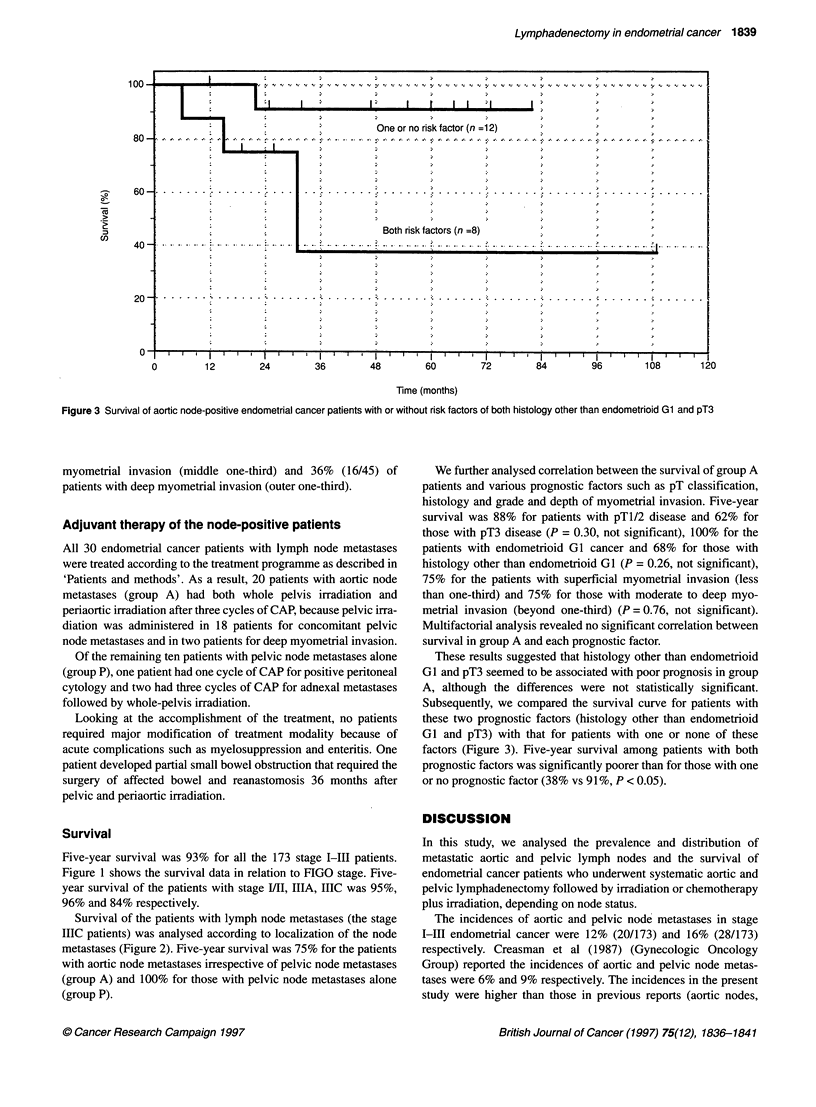

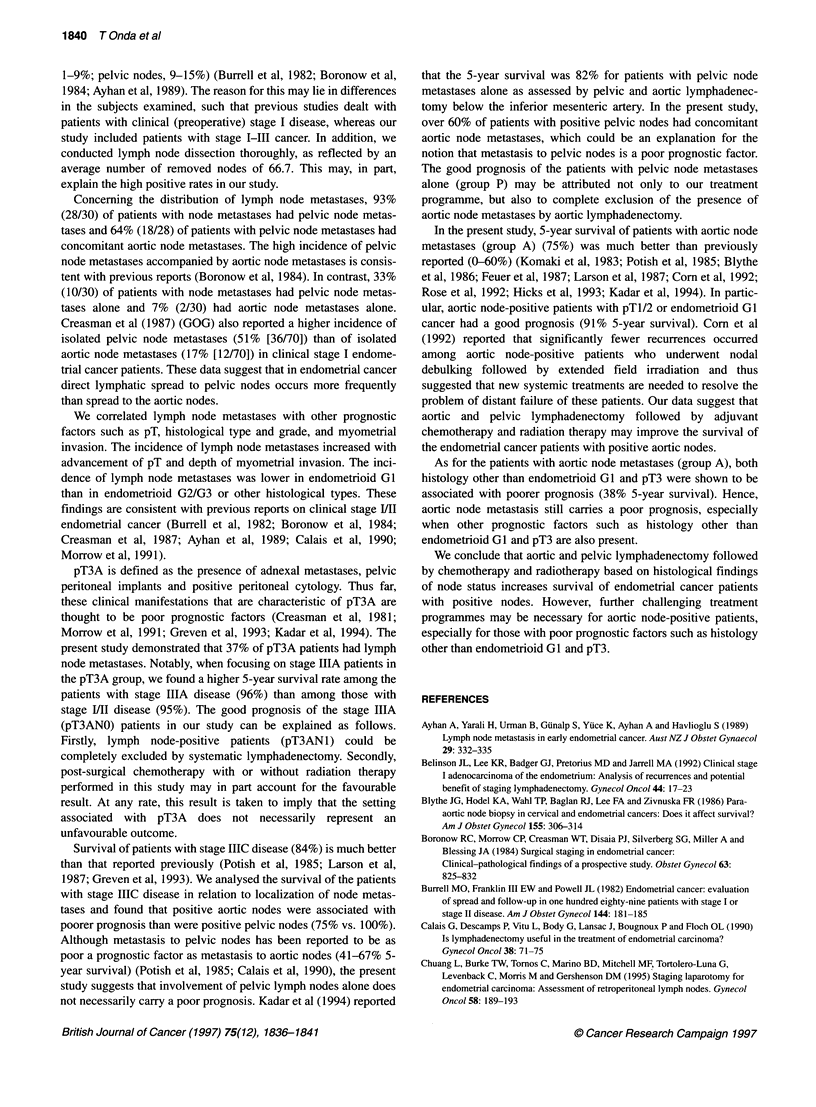

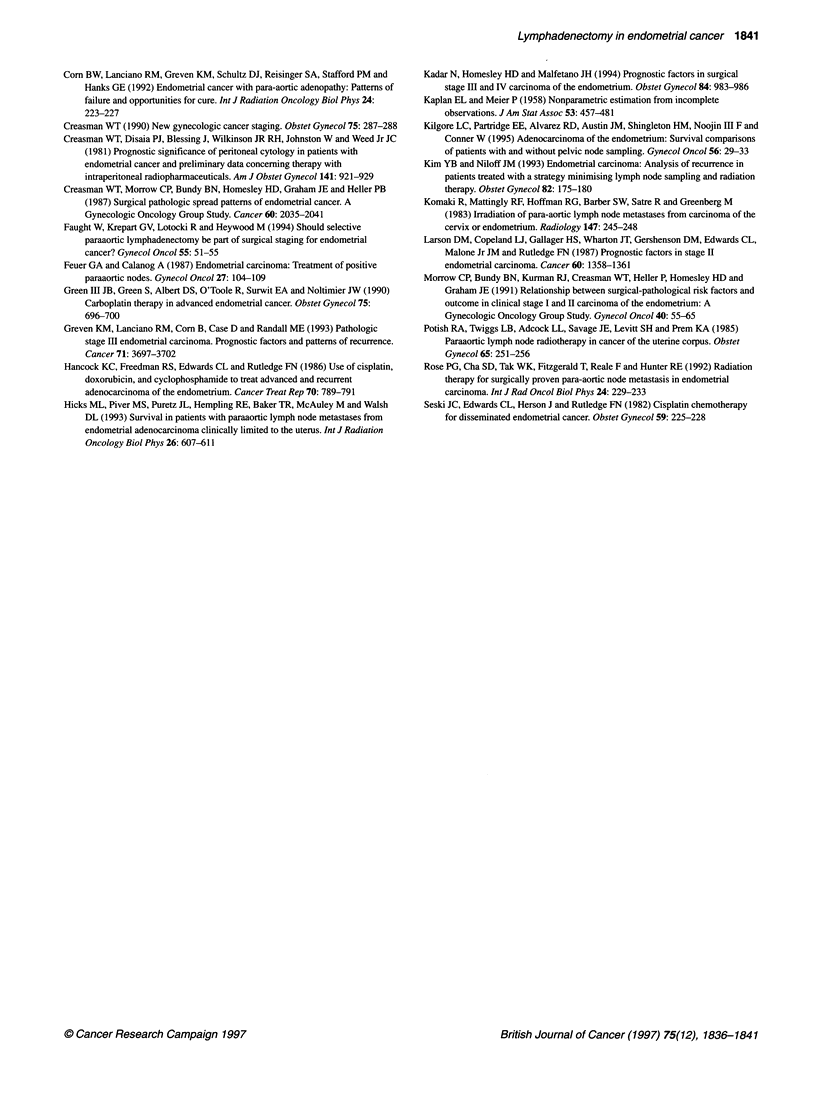


## References

[OCR_00775] Ayhan A., Yarali H., Urman B., Günalp S., Yüce K., Ayhan A., Havlioglu S. (1989). Lymph node metastasis in early endometrium cancer.. Aust N Z J Obstet Gynaecol.

[OCR_00780] Belinson J. L., Lee K. R., Badger G. J., Pretorius R. G., Jarrell M. A. (1992). Clinical stage I adenocarcinoma of the endometrium--analysis of recurrences and the potential benefit of staging lymphadenectomy.. Gynecol Oncol.

[OCR_00785] Blythe J. G., Hodel K. A., Wahl T. P., Baglan R. J., Lee F. A., Zivnuska F. R. (1986). Para-aortic node biopsy in cervical and endometrial cancers: does it affect survival?. Am J Obstet Gynecol.

[OCR_00790] Boronow R. C., Morrow C. P., Creasman W. T., Disaia P. J., Silverberg S. G., Miller A., Blessing J. A. (1984). Surgical staging in endometrial cancer: clinical-pathologic findings of a prospective study.. Obstet Gynecol.

[OCR_00797] Burrell M. O., Franklin E. W., Powell J. L. (1982). Endometrial cancer: evaluation of spread and follow-up one hundred eighty-nine patients with Stage I or Stage II disease.. Am J Obstet Gynecol.

[OCR_00802] Calais G., Descamps P., Vitu L., Body G., Lansac J., Bougnoux P., Le Floch O. (1990). Is lymphadenectomy useful in the treatment of endometrial carcinoma?. Gynecol Oncol.

[OCR_00807] Chuang L., Burke T. W., Tornos C., Marino B. D., Mitchell M. F., Tortolero-Luna G., Levenback C., Morris M., Gershenson D. M. (1995). Staging laparotomy for endometrial carcinoma: assessment of retroperitoneal lymph nodes.. Gynecol Oncol.

[OCR_00817] Corn B. W., Lanciano R. M., Greven K. M., Schultz D. J., Reisinger S. A., Stafford P. M., Hanks G. E. (1992). Endometrial cancer with para-aortic adenopathy: patterns of failure and opportunities for cure.. Int J Radiat Oncol Biol Phys.

[OCR_00824] Creasman W. T., Disaia P. J., Blessing J., Wilkinson R. H., Johnston W., Weed J. C. (1981). Prognostic significance of peritoneal cytology in patients with endometrial cancer and preliminary data concerning therapy with intraperitoneal radiopharmaceuticals.. Am J Obstet Gynecol.

[OCR_00831] Creasman W. T., Morrow C. P., Bundy B. N., Homesley H. D., Graham J. E., Heller P. B. (1987). Surgical pathologic spread patterns of endometrial cancer. A Gynecologic Oncology Group Study.. Cancer.

[OCR_00823] Creasman W. T. (1990). New gynecologic cancer staging.. Obstet Gynecol.

[OCR_00836] Faught W., Krepart G. V., Lotocki R., Heywood M. (1994). Should selective paraaortic lymphadenectomy be part of surgical staging for endometrial cancer?. Gynecol Oncol.

[OCR_00841] Feuer G. A., Calanog A. (1987). Endometrial carcinoma: treatment of positive paraaortic nodes.. Gynecol Oncol.

[OCR_00845] Green J. B., Green S., Alberts D. S., O'Toole R., Surwit E. A., Noltimier J. W. (1990). Carboplatin therapy in advanced endometrial cancer.. Obstet Gynecol.

[OCR_00850] Greven K. M., Lanciano R. M., Corn B., Case D., Randall M. E. (1993). Pathologic stage III endometrial carcinoma. Prognostic factors and patterns of recurrence.. Cancer.

[OCR_00855] Hancock K. C., Freedman R. S., Edwards C. L., Rutledge F. N. (1986). Use of cisplatin, doxorubicin, and cyclophosphamide to treat advanced and recurrent adenocarcinoma of the endometrium.. Cancer Treat Rep.

[OCR_00860] Hicks M. L., Piver M. S., Puretz J. L., Hempling R. E., Baker T. R., Mcauley M., Walsh D. L. (1993). Survival in patients with paraaortic lymph node metastases from endometrial adenocarcinoma clinically limited to the uterus.. Int J Radiat Oncol Biol Phys.

[OCR_00866] Kadar N., Homesley H. D., Malfetano J. H. (1994). Prognostic factors in surgical stage III and IV carcinoma of the endometrium.. Obstet Gynecol.

[OCR_00873] Kilgore L. C., Partridge E. E., Alvarez R. D., Austin J. M., Shingleton H. M., Noojin F., Conner W. (1995). Adenocarcinoma of the endometrium: survival comparisons of patients with and without pelvic node sampling.. Gynecol Oncol.

[OCR_00877] Kim Y. B., Niloff J. M. (1993). Endometrial carcinoma: analysis of recurrence in patients treated with a strategy minimizing lymph node sampling and radiation therapy.. Obstet Gynecol.

[OCR_00882] Komaki R., Mattingly R. F., Hoffman R. G., Barber S. W., Satre R., Greenberg M. (1983). Irradiation of para-aortic lymph node metastases from carcinoma of the cervix or endometrium. Preliminary results.. Radiology.

[OCR_00887] Larson D. M., Copeland L. J., Gallager H. S., Wharton J. T., Gershenson D. M., Edwards C. L., Malone J. M., Rutledge F. N. (1987). Prognostic factors in stage II endometrial carcinoma.. Cancer.

[OCR_00892] Morrow C. P., Bundy B. N., Kurman R. J., Creasman W. T., Heller P., Homesley H. D., Graham J. E. (1991). Relationship between surgical-pathological risk factors and outcome in clinical stage I and II carcinoma of the endometrium: a Gynecologic Oncology Group study.. Gynecol Oncol.

[OCR_00898] Potish R. A., Twiggs L. B., Adcock L. L., Savage J. E., Levitt S. H., Prem K. A. (1985). Paraaortic lymph node radiotherapy in cancer of the uterine corpus.. Obstet Gynecol.

[OCR_00903] Rose P. G., Cha S. D., Tak W. K., Fitzgerald T., Reale F., Hunter R. E. (1992). Radiation therapy for surgically proven para-aortic node metastasis in endometrial carcinoma.. Int J Radiat Oncol Biol Phys.

[OCR_00908] Seski J. C., Edwards C. L., Herson J., Rutledge F. N. (1982). Cisplatin chemotherapy for disseminated endometrial cancer.. Obstet Gynecol.

